# A Turn-on Fluorescence Sensor for Heparin Detection Based on a Release of Taiwan Cobra Cardiotoxin from a DNA Aptamer or Adenosine-Based Molecular Beacon

**DOI:** 10.3390/molecules23020460

**Published:** 2018-02-19

**Authors:** Yi-Jun Shi, Liang-Jun Wang, Yuan-Chin Lee, Chia-Hui Huang, Wan-Ping Hu, Long-Sen Chang

**Affiliations:** 1 Institute of Biomedical Sciences, National Sun Yat-Sen University, Kaohsiung 804, Taiwan; a786514@gmail.com (Y.-J.S.); bringwang42@gmail.com (L.-J.W.); d042050010@student.nsysu.edu.tw (Y.-C.L.) nikki781016@hotmail.com (C.-H.H.); 2 Department of Biotechnology, Kaohsiung Medical University, Kaohsiung 807, Taiwan; wphu@kmu.edu.tw

**Keywords:** aptamer, molecular beacon, cardiotoxin, heparin, turn-on fluorescence sensor

## Abstract

This study presents two sensitive fluorescent assays for sensing heparin on the basis of the electrostatic interaction between heparin and *Naja naja atra* cardiotoxin 3 (CTX3). Owing to CTX3-induced folded structure of an adenosine-based molecular beacon (MB) or a DNA aptamer against CTX3, a reduction in the fluorescent signal of the aptamer or MB 5′-end labeled with carboxyfluorescein (FAM) and 3′-end labeled with 4-([4-(dimethylamino)phenyl]azo)-benzoic acid (DABCYL) was observed upon the addition of CTX3. The presence of heparin and formation of the CTX3–heparin complex caused CTX3 detachment from the MB or aptamer, and restoration of FAM fluorescence of the 5′-FAM-and-3′-DABCYL-labeled MB and aptamer was subsequently noted. Moreover, the detection of heparin with these CTX3-aptamer and CTX3-MB sensors showed high sensitivity and selectivity toward heparin over chondroitin sulfate and hyaluronic acid regardless of the presence of plasma. The limit of detection for heparin in plasma was determined to be 16 ng/mL and 15 ng/mL, respectively, at a signal-to-noise ratio of 3. This study validates the practical utility of the CTX3-aptamer and CTX3-MB systems for determining the concentration of heparin in a biological matrix.

## 1. Introduction

Heparin, which consists of a trisulfated repeating disaccharide unit, is widely used as an anticoagulant or antithrombotic agent during clinical procedures such as cardiovascular surgery. An overdose of heparin can cause adverse effects such as hemorrhages and thrombocytopenia [1,2]. The proper therapeutic concentration range of heparin in plasma has been found to be 17–67 μM during cardiovascular surgery and 1.7–10 μM in the postoperative period [3]. Therefore, the development of rapid and sensitive methods for the quantification of heparin in blood is important for reducing side effects of heparin. Measurements of activated clotting time or activated partial thromboplastin time are the traditional procedures for heparin detection [2,4]. These methods are not sufficiently sensitive or accurate for detection of heparin because of their lack of specificity and potential interference from other factors [5]. Thus, a number of methods for the quantitative detection of heparin has been developed including a fluorescent method [6], a colorimetric assay [7,8], and an electrochemical method [9]. These methods offer reasonable sensitivity and specificity, but some are complicated and time-consuming. Many reported methods are based on complicated nano-organic hybrid systems or “turn-off” ones. In accordance with the notion that a “turn-on” method can effectively eliminate the false results caused by various uncertain factors, turn-on strategies are more reliable than turn-off ones [10]. Therefore, the development of sensitive turn-on fluorescence probes for heparin detection is still highly desired.

Cardiotoxins (CTXs), a group of major venom polypeptides ~60 amino acid residues long that are present abundantly in the elapid family of snakes, have well-pronounced pharmacological effects including hemolysis, cytotoxicity, and depolarization of muscles [11]. Owing to their positively charged residues, CTXs are believed to damage cells by their ability to interact with anionic lipids or negatively charged oligosaccharides on the plasma membrane [12,13,14,15]. Sue et al. [13] found that CTXs bind to heparin and heparin-derived hexasaccharide. In line with this finding, Chiou et al. [16] and Kao et al. [17] reported that heparin inhibits the membrane-damaging activity of *Naja naja atra* cardiotoxin 3 (CTX3). Recent studies revealed that CTX3 can induce a folded structure of an adenosine (A)-based molecular beacon (MB) [18]. Because the CTX3-bound MB conformation brings 5′-end-attached carboxyfluorescein (FAM) and 3′-end-attached 4-([4-(dimethylamino)phenyl]azo)-benzoic acid (DABCYL) into close proximity, the binding of CTX3 to the MB results in quenching of FAM fluorescence. Likewise, our previous studies have shown that the binding of CTX3 induces conformational changes of 5′-FAM-and-3′-DABCYL-labeled DNA aptamers against CTX3 and thereby causes a reduction in FAM fluorescence [19]. As for the findings that CTX3 can bind to heparin [13,16,17], the fluorescence intensity of an aptamer or MB may increase when CTX3 is removed from a complex with an aptamer or MB by heparin. Accordingly, we hypothesized that the pair of CTX3 with an aptamer or MB may be a useful sensor system for the detection of heparin.

## 2. Results and Discussion

**Figure 1:**
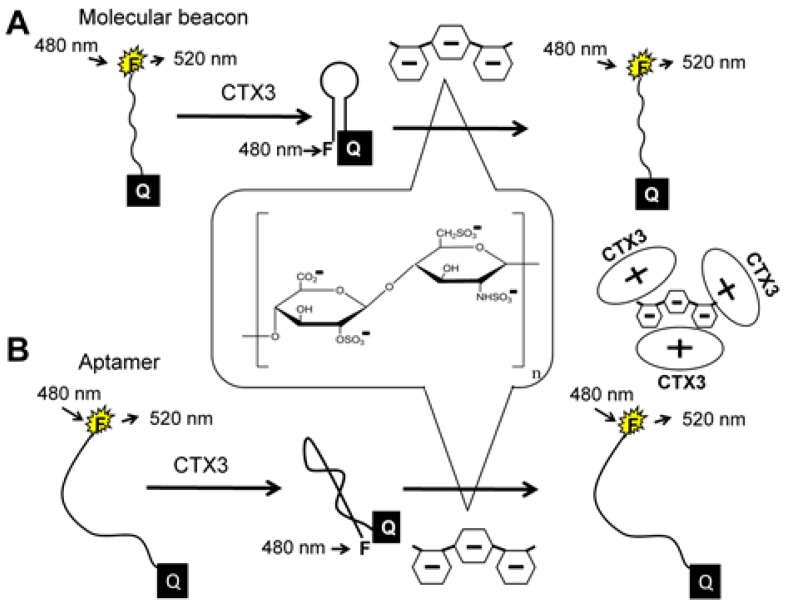
Turn-on fluorescence detection of heparin based on competitive binding (**A**) between heparin and molecular beacon (MB) to CTX3 or (**B**) between heparin and aptamer to CTX3.

The principle of heparin detection with CTX3 is illustrated in Figure 1. Our recent studies proved that the binding of CTX3 to a 5′-FAM-and-3′-DABCYL-labeled aptamer or A_20_-MB-A_20_ brings FAM and DABCYL into close proximity, leading to quenching of FAM fluorescence of the aptamer and MB [18,19]. The amino groups of CTX3 may interact with negatively charged sulfate and carboxylate groups of heparin through electrostatic interaction [13,17]. When CTX3 is freed from an aptamer or MB by heparin, a reversal increase in FAM fluorescence takes place. Accordingly, CTX3-induced fluorescence quenching of an aptamer and MB can be employed to detect heparin by turning on FAM fluorescence.

**Figure 2:**
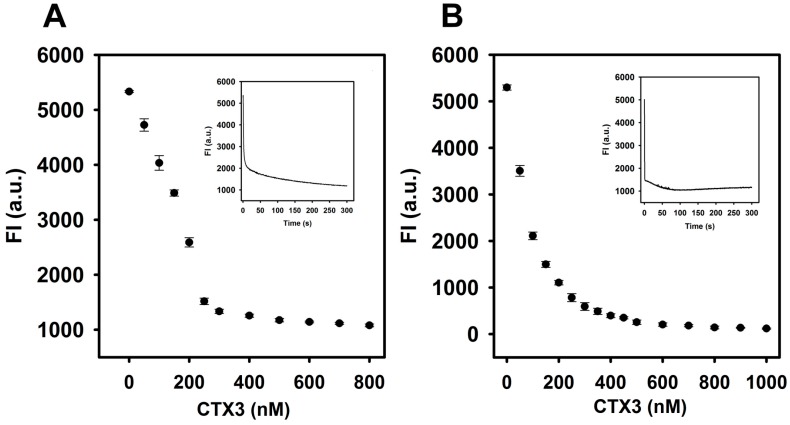
Fluorescence intensity at 520 nm of aptamer and A_12_-MB-A_12_ was reduced by titrating with CTX3: (**A**) FAM/DABCYL-labeled aptamer (20 nM) and (**B**) A_12_-MB-A_12_ (10 nM) were titrated with indicated CTX3 concentrations. The experiments were conducted at 30 °C. (Inset of **A**) time course measurement of FAM intensity (520 nm) of aptamer upon the addition of 300 nM CTX3; (Inset of **B**) time course measurement of FAM intensity of MB upon the addition of 200 nM CTX3.

**Figure 3:**
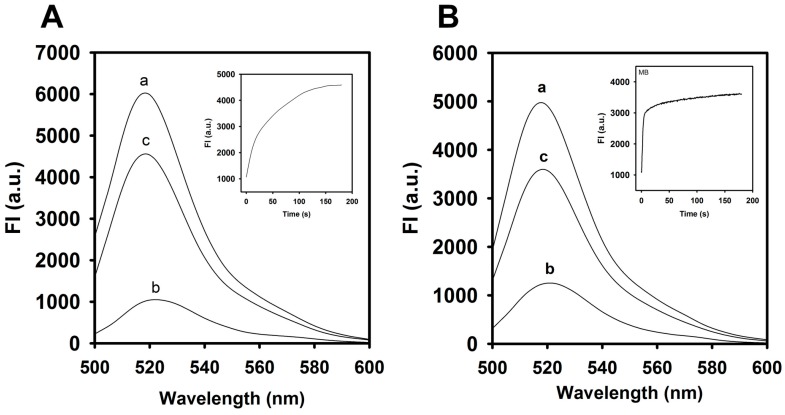
Fluorescence spectra of aptamer and A_12_-MB-A_12_ in the absence or presence of CTX3 and heparin: The incubation time of CTX3-aptamer or CTX3-MB sensors with heparin was 5 min. (**A**) fluorescence spectra of (line a) 20 nM aptamer, (line b, line c) 20 nM aptamer and 300 nM CTX3 in the absence and presence of 1.8 μg/mL heparin. (Inset) Time course measurement of fluorescence intensity (520 nm) of a solution containing 20 nM aptamer and 300 nM CTX3 upon the addition of 1.8 μg/mL heparin. (**B**) fluorescence spectra of (line a) 10 nM MB, (line b, line c) 10 nM MB and 200 nM CTX3 in the absence and presence of 1.8 μg/mL heparin. (Inset) Time course measurement of fluorescence intensity (520 nm) of a solution containing 10 nM MB and 200 nM CTX3 upon the addition of 1.8 μg/mL heparin.

As shown in Figure 2, the FAM fluorescence intensity of the aptamer or MB was reduced by the addition of CTX3 and decreased maximally within 300 s at 520 nm. The fluorescence quenching of the aptamer and MB reached a saturation level after addition of 400 nM CTX3. Compared to the absence of CTX3, the binding of CTX3 to the aptamer or MB resulted in more than 80% quenching of FAM fluorescence. As shown in Figure 3, a remarkable increase in the fluorescence of FAM occurred after 1.8 μg/mL heparin (0.1 μM, MW of heparin ~18,000 Da) was incubated with a solution of the CTX3–aptamer or CTX3–MB complex. These results revealed that heparin was capable of detaching CTX3 from the aptamer or MB. The inset of Figure 3 shows that the fluorescence turn-on assay for heparin requires at least 3 min to achieve a maximal recovery in the fluorescence of FAM. Thus, the incubation time for detecting heparin was optimized (5 min). 

**Figure 4:**
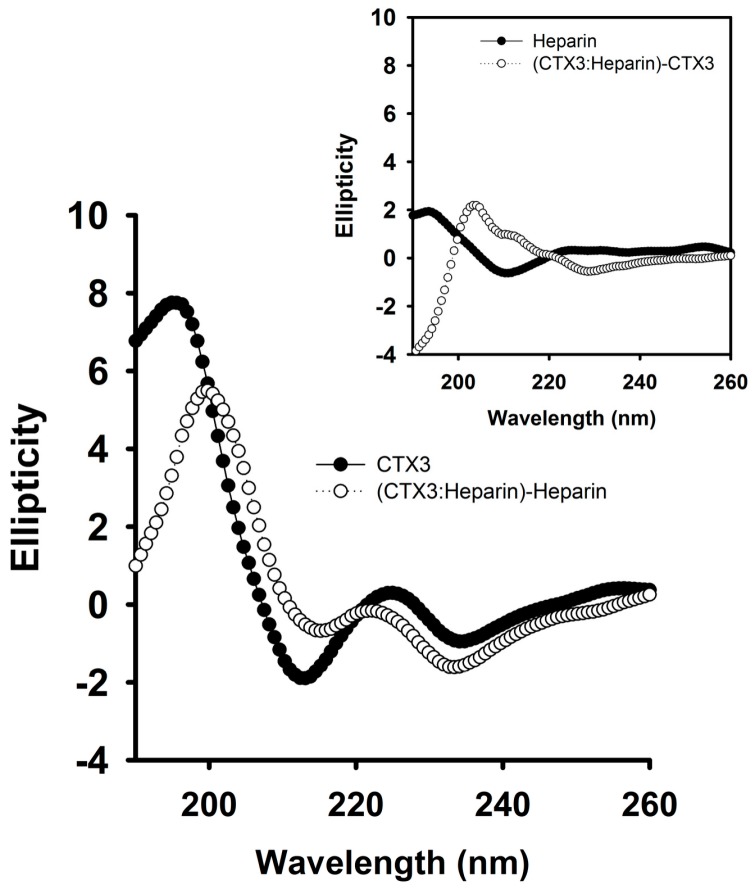
The CD spectra of CTX3 and CTX3-heparin complexes. As shown in Figure 3, the addition of 1.8 μg/mL heparin notably detached CTX3 (300 nM) from aptamer. Thus, the CD spectra were measured at a CTX3 concentration of 300 μM and a heparin concentration of 100 μM (1800 μg/mL, MW of heparin ~18,000) in 10 mM 4-(2-hydroxyethyl)piperazine-1-ethanesulfonic acid (HEPES) (pH 8.0). The CD spectrum of CTX3-heparin complexes was subtracted by that of heparin. (Inset) The CD spectrum of CTX3-heparin complexes was subtracted by that of CTX3.

**Figure 5:**
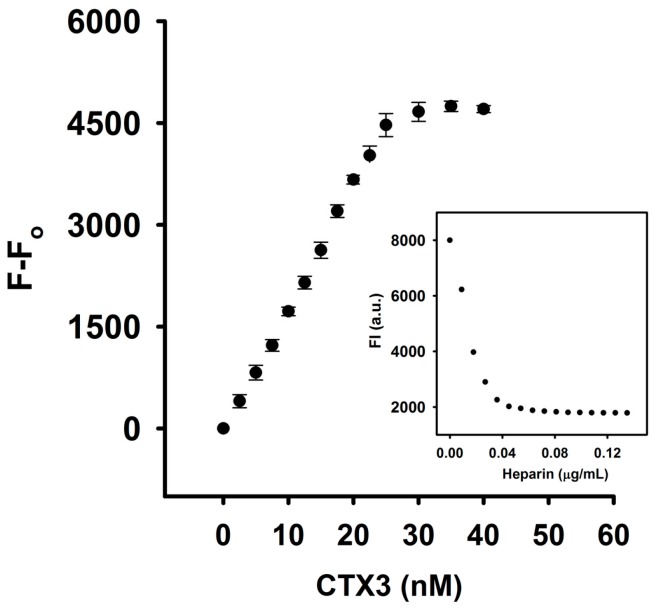
Replacement of heparin-bound FITC-CTX3 by CTX3. FITC-CTX3 (71.4 nM) dissolved in 10 mM HEPES (pH 8.0) were incubated with 0.036 μg/mL heparin for 1 h, and increasing concentrations of unlabeled CTX3 were then added. Replacement of heparin-bound FITC-CTX3 by unlabeled CTX3 led to a restoration in fluorescence intensity of FITC-CTX3. (Inset) Maximal reduction in fluorescence intensity of FITC-CTX3 (71.4 nM) was noted when 0.08 μg/mL heparin was added, while the addition of 0.036 μg/mL heparin reduced the fluorescence intensity of FITC-CTX3 to approximately 95% of the maximum.

**Figure 6:**
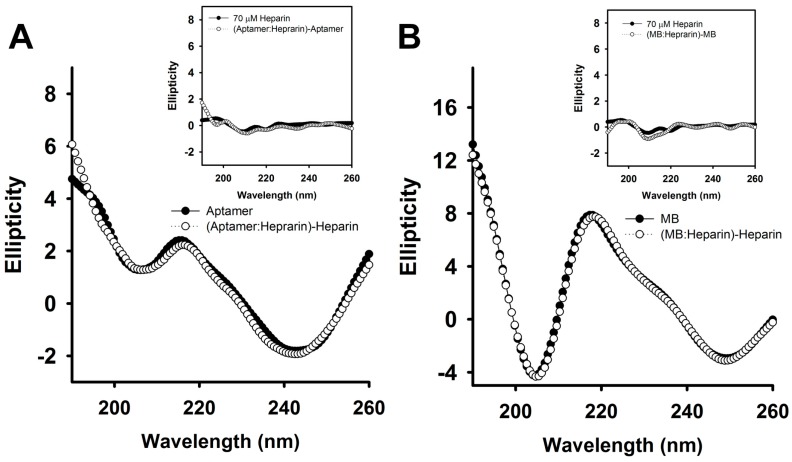
The CD spectra of aptamer and MB in the absence or presence of heparin. As shown in Figure 9A,B, the addition of 0.36 μg/mL (20 nM, MW ~ 18,000) notably detached CTX3 from aptamer (20 nM) or MB (10 nM). Thus, two-fold molar excess of heparin over aptamer and MB was used. The CD spectra were measured at an aptamer concentration of 35 μM, a MB concentration of 35 μM and a heparin concentration of 70 μM in 10 mM HEPES (pH 8.0). (**A**) the CD spectrum of aptamer-heparin mixtures was subtracted by that of heparin. (Inset) The CD spectrum of aptamer-heparin mixtures was subtracted by that of aptamer; (**B**) the CD spectrum of MB-heparin mixtures was subtracted by that of heparin. (Inset) The CD spectrum of MB-heparin mixtures was subtracted by that of MB.

To further prove that heparin detached CTX3 from aptamer and MB, the interaction of heparin with CTX3, aptamer and MB was analyzed. As shown in Figure 4, CD measurement showed that heparin induced structural change of CTX3 and/or CTX3 induced structural change of heparin, indicating that heparin bound with CTX3. To investigate the interaction of CTX3 with heparin, replacement of heparin-bound FITC-CTX3 by unlabeled CTX3 was conducted. As shown in Figure 5, the fluorescence intensity of FITC-CTX3 was reduced by titrating with heparin, and the maximal reduction in fluorescence intensity of FITC-CTX3 was noted when 0.08 μg/mL heparin was added. This reflected that changes in conformation of FITC-CTX3 occurred upon binding with heparin. Gradual restoration of fluorescence intensity was noted upon the addition of unlabeled CTX3, indicating competitive replacement of FITC-CTX3 from heparin. The binding constant calculated from the changes in fluorescence intensity showed that the dissociation constants of CTX3 for heparin were 0.17 ± 0.03 μM. On the other hand, the addition of heparin did not change fluorescence intensity of either 5′-FAM-and-3′-DABCYL-labeled aptamer or MB (supplementary Figure S1). Moreover, heparin marginally altered CD spectra of aptamer and MB or *vice versa* (Figure 6). These results again supported that heparin detached CTX3 from aptamer and MB, leading to the fluorescence recovery of CTX3-aptamer and CTX3-MB sensors.

**Figure 7:**
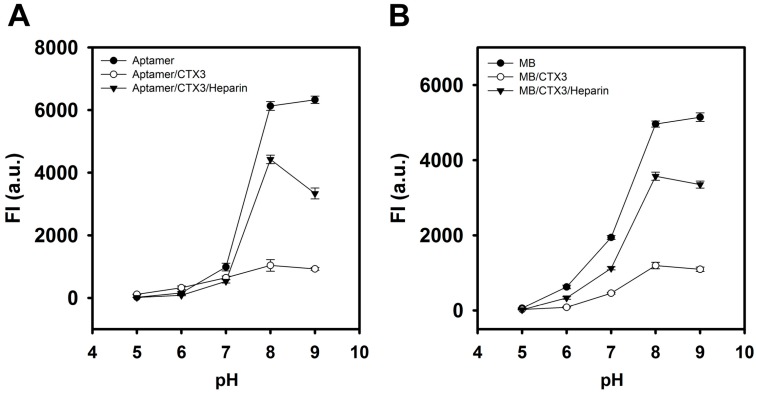
Effect of pH on the fluorescence recovery of (**A**) CTX3-aptamer and (**B**) CTX3-MB sensors with the addition of heparin. (**A**) the used aptamer, CTX3, and heparin concentrations were 20 nM, 300 nM, and 1.8 μg/mL, respectively; (**B**) the used MB, CTX3, and heparin concentration were 10 nM, 200 nM, and 1.8 μg/mL, respectively.

The effect of pH on the FAM fluorescence of aptamer, MB, CTX3-aptamer complexes, and CTX3-MB complexes was also investigated in the pH range 5–9. As shown in Figure 7, the fluorescence intensity of aptamer and MB alone reached its maximum at pH 8–9. The addition of CTX3 caused a maximum reduction in FAM fluorescence of aptamer and MB at pH 8–9. Upon the addition of heparin, maximal fluorescence recovery of CTX3-aptamer and CTX3-MB complexes was noted at pH 8. Thus, pH 8 was selected for conducting the experiments in the present study.

**Figure 8:**
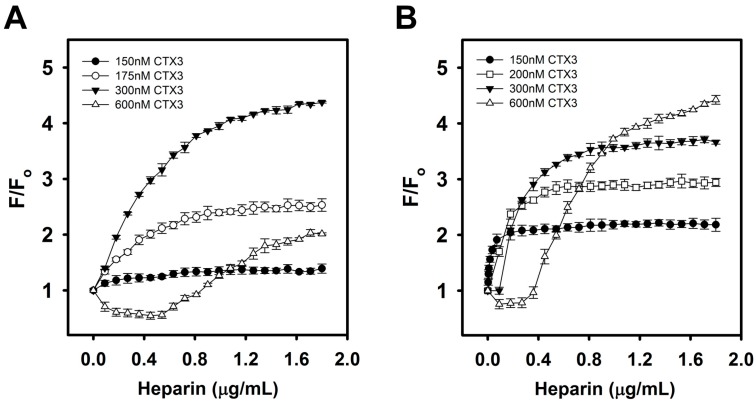
Effect of varying CTX3 concentrations on the turn-on fluorescence of CTX3-aptamer or CTX3-MB sensors upon the titration of indicated heparin concentrations: (**A**) aptamer and (**B**) MB were incubated with varying CTX3 concentrations for 5 min, and then CTX3-aptamer or CTX3-MB sensors were titrated with the indicated heparin concentrations.

Given that the recovery of FAM fluorescence is based on the ability of heparin to detach CTX3 from the aptamer or MB, it is plausible that the CTX3 concentration affected not only the fluorescence intensity but also the sensitivity of the assay. The effect of CTX3 concentration on the ability of the CTX3-aptamer and CTX3-MB sensors to detect heparin was therefore investigated. Figure 8 shows that different fluorescence recovery efficiency rates were observed upon the addition of different amounts of CTX3 to the systems. The fluorescence recovery efficiency gradually increased as the CTX3 concentration increased in the range from 150 to 300 nM. The concentration-dependent effect of heparin on fluorescence recovery efficiency started to change when the CTX3 concentration exceeded 300 nM. The increasing fluorescence recovery efficiency up to a CTX3 concentration of 300 nM was attributed to greater amounts of CTX3 being bound by the aptamer or MB when more CTX3 was present, leading to a remarkable decrease in the fluorescence intensity (Fo) of the CTX3-aptamer and CTX3-MB systems and an increase in the recovery of fluorescence intensity in the presence of heparin. Nevertheless, the presence of more CTX3 means that more heparin is needed to detach CTX3 from the aptamer or MB to form the CTX3–heparin complex and to recover the fluorescence. Consequently, an increase in the required heparin concentration is noted when the CTX3 concentration increases. As shown in Figure 8, the FAM fluorescence of the aptamer–600 nM CTX3 pair, MB–300 nM CTX3 pair and MB–600 nM CTX3 pair could not effectively respond to a low heparin concentration. Although the difference between the blank and sample signals in the MB–300 nM CTX3 pair was greater than that in the MB–200 nM CTX3 pair, the inability to detect low heparin concentration will be undesirable because it leads to the loss of sensitivity of concentration-dependent heparin detection. After we take these conditions into consideration, the aptamer–300 nM CTX3 and MB–200 nM CTX3 pairs were selected for the heparin titration experiments.

**Figure 9:**
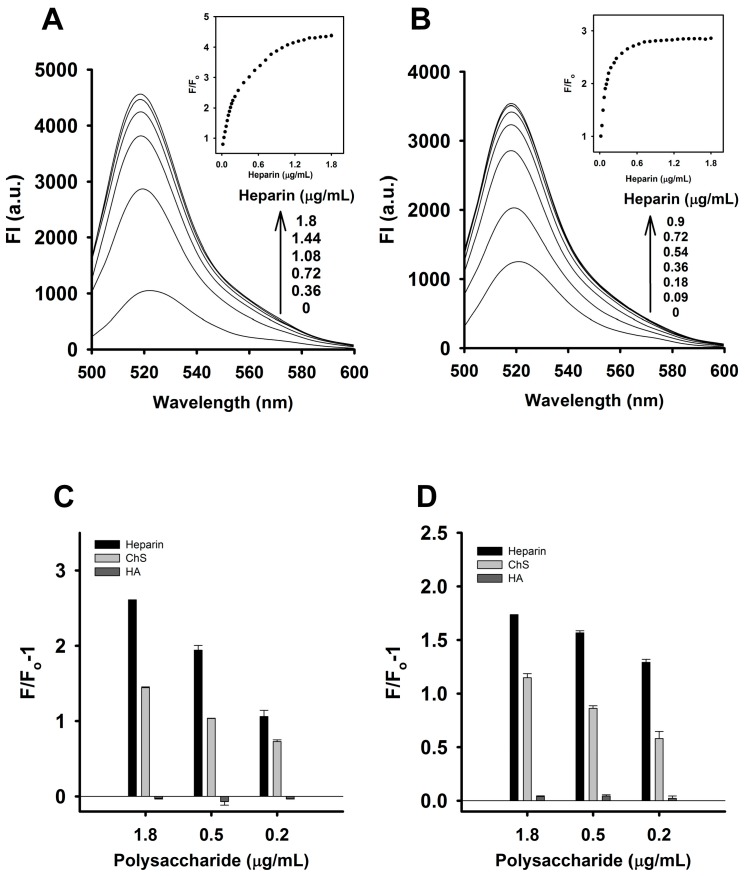
Effect of heparin on fluorescence intensity at 520 nm of CTX3-aptamer and CTX3-MB sensors: The arrows indicate the signal changes with increasing heparin concentrations. (**A**) fluorescence spectra of a solution of 20 nM aptamer and 300 nM CTX3 in the presence of indicated heparin concentrations. (Inset) The relative fluorescence intensity plotted against various heparin concentrations. (**B**) fluorescence spectra of a solution of 10 nM MB and 200 nM CTX3 in the presence of indicated heparin concentrations. (Inset) The relative fluorescence intensity plotted against various heparin concentrations. Fo and F corresponded to the fluorescence intensity of FAM at 520 nm in the absence or presence of heparin, respectively. Changes in the fluorescence intensity of (**C**) CTX3-aptamer and (**D**) CTX3-MB sensors upon the addition of heparin, chondroitin sulfate (ChS) and hyaluronic acid (HA). Fo and F corresponded to the fluorescence intensity of FAM at 520 nm as measured from CTX3-aptamer and CTX3-MB sensors in the absence or presence of indicated heparin, ChS and HA concentrations.

The sensitivity of the CTX3-aptamer and CTX3-MB sensors for detecting heparin was determined under the optimal conditions described above. The fluorescence intensity of the CTX3-aptamer and CTX3-MB sensors increased as the heparin concentration increased, indicating that the addition of heparin to the CTX3-aptamer or CTX3-MB system caused CTX3 to desorb from the aptamer and MB. The fluorescence intensity of the aptamer increased with the increasing heparin concentration as shown in Figure 9A, where a linear plot (Y = 11.08X + 0.77, R^2^ = 0.994) of the fluorescence intensity of the aptamer at 520 nm (at the maximum fluorescence emission peak) was obtained, with a linear range of 18–180 ng/mL. The limit of detection (LOD) was determined by means of the equation LOD = 3δ/S, where δ is the standard deviation of the blank assays (*n* = 10) and S is the slope of the calibration plot. The LOD of heparin in this assay reached 8.7 ng/mL. Likewise, the fluorescence intensity of the MB increased with the increasing heparin concentration (Figure 9B). The inset in Figure 9B depicts the relation between the fluorescence intensity and the heparin concentration. A plot of F/Fo against the heparin concentration indicated that the assay has a linear range of 18–108 ng/mL, in which the calibration equation was Y= 12.95X + 0.77 and the correlation coefficient (R^2^) was 0.993. The LOD was found to be 12 ng/mL.

To evaluate the selectivity of the CTX3-aptamer and CTX3-MB sensors for detecting heparin, the response of the CTX3-aptamer or CTX3-MB to two other sulfated glycosaminoglycans, chondroitin sulfate (ChS) and hyaluronic acid (HA) was investigated. As shown in Figure 9C,D, the CTX3-aptamer or CTX3-MB sensor manifested a marginal change in fluorescence intensity in the presence of HA, whereas the addition of ChS restored the fluorescence intensity of the CTX3-aptamer and CTX3-MB sensors. Nevertheless, the ability of ChS to recover FAM fluorescence of CTX3-aptamer and CTX3-MB sensors was lower than that of heparin. These results may be mainly explained as follows: the negative charge density per repeat unit of HA and ChS is lower than that in heparin. HA has only one carboxyl group, and two negatively charged groups (sulfate and carboxylate) per unit are present in ChS. In contrast, heparin possesses three sulfate groups and one carboxylate per repeat unit. These results indicate that the CTX3-aptamer and CTX3-MB sensors have higher selectivity for heparin.

**Figure 10:**
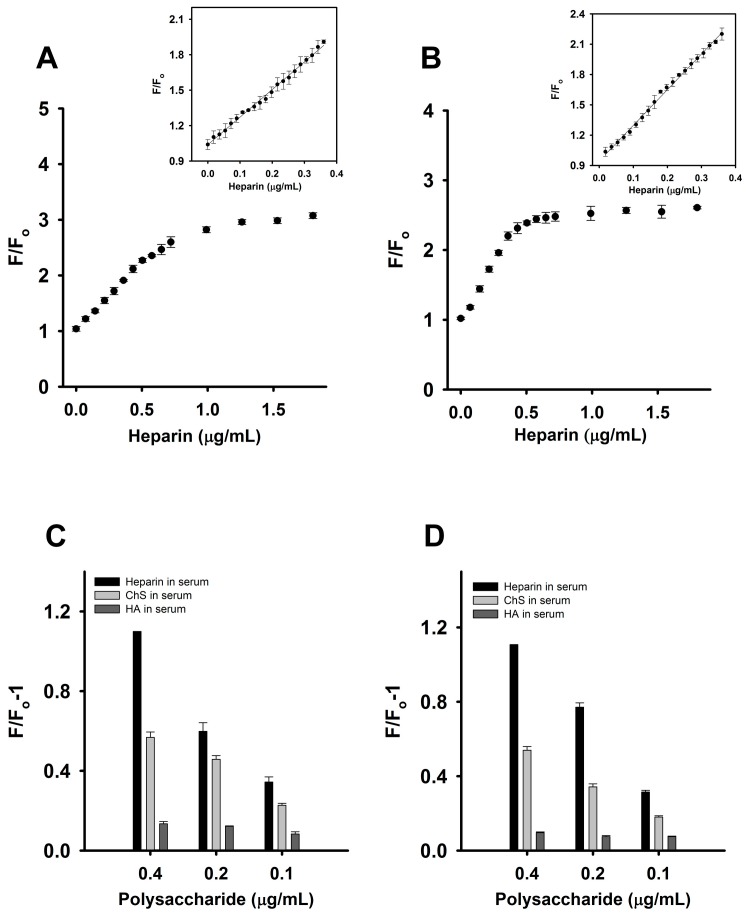
Determination of heparin in plasma samples: Fo and F corresponded to the fluorescence intensity of FAM at 520 nm in the absence or presence of heparin, respectively. (**A**) changes in the fluorescence intensity of solutions of 20 nM aptamer and 300 nM CTX3 in the presence of heparin-spiked plasma samples. (Inset) A plot of the F/Fo value versus the spiked concentrations of heparin; (**B**) changes in the fluorescence intensity of solutions of 10 nM MB and 200 nM CTX3 in the presence of heparin-spiked plasma samples. (Inset) A plot of the F/Fo value versus the spiked concentrations of heparin. Changes in the fluorescence intensity of (**C**) CTX3-aptamer and (**D**) CTX3-MB sensors upon the addition of heparin-, ChS- and HA-spiked plasma samples. Fo and F corresponded to the fluorescence intensity at 520 nm of FAM as measured from CTX3-aptamer and CTX3-MB sensor in the absence or presence of plasma samples spiked with indicated heparin, ChS and HA concentrations.

To demonstrate a practical application of this fluorescence turn-on assay for heparin, detection of this analyte in human serum was studied next. The FAM fluorescence intensity of the CTX3-aptamer and CTX3-MB sensors marginally increased upon the addition of a small volume (1–5 μL) of serum without spiking with heparin (data not shown). Thus, the sensors may allow researchers to directly detect heparin in a small volume of a heparin-containing serum sample. After that, human serum samples were spiked with different concentrations of heparin, and then 1 μL of the heparin-containing serum was directly added into a 2 mL assay solution. The final dilution of serum protein of 0.05% was used in the assay method. The fluorescence intensity of the CTX3-MB and CTX3-aptamer sensors increased with the increasing heparin concentration as shown in Figure 10A,B. Together with the notion that 1 μL of serum without spiking with heparin did not appreciably affect the fluorescence of CTX3-aptamer and CTX3-MB sensors, these results indicated that the action of heparin on the detachment of CTX3 from aptamer and MB was exclusively responsible for the restoration in FAM fluorescence of CTX3-aptamer and CTX3-MB sensors. A linear plot (Y= 2.34X + 1.04, R^2^ = 0.996) of the fluorescence intensity of the aptamer at 520 nm was obtained (Figure 10A), with a linear range of 18–360 ng/mL, and the LOD of heparin in this assay reached 16 ng/mL. Likewise, the fluorescence intensity of the MB increased with the increasing heparin concentration (Figure 10B). A plot of F/Fo against the heparin concentration showed that the assay has a linear range of 18–360 ng/mL, in which the calibration equation is Y= 3.52X + 0.95 and the correlation coefficient (R^2^) is 0.997. The LOD for heparin in this assay was found to be 15 ng/mL. The response of the CTX3-aptamer and CTX3-MB sensors to HA and ChS in serum samples was also evaluated. As presented in Figure 10C,D, compared to ChS and HA, the two detection systems showed a superior response to heparin in serum samples. Therefore, the CTX3-aptamer and CTX3-MB sensors are promising fluorescence probes for the detection of heparin in the serum matrix.

## 3. Materials and Methods 

Cardiotoxin 3 (CTX3) was isolated from the venom of *N. naja atra* (Taiwan cobra) according to the procedure described in Lin et al. [20]. Fluorescein isothiocyanate (FITC), heparin (sodium salt; MW ~ 18,000) from porcine intestinal mucosa, chondroitin sulfate (sodium salt) from bovine trachea and hyaluronic acid (sodium salt) from bovine vitreous humor were purchased from Sigma-Aldrich Inc. (St. Louis, MO, USA), and 5′-FAM/3′-DABCYL-labeled A_12_-CATCATAGTCGAGTGTCCAGGG-A_12_ DNA MB and 5′-FAM/3′-DABCYL-labeled DNA aptamer against CTX3 with sequence of 5′-GCGAGGTGTTCGAGAGTTAGGGGCGACATGACCAAACGTT-3′ were synthesized from Neogene Biomedicals Corporation (City, Taiwan). Our previous studies have shown that CTX isotoxins similarly bind with the MB and aptamer [18,19]. Although CTX3 is not commercial available, CTX isotoxins can be obtained from Accurate Chemical & Scientific Co. (Westbury, NY, USA) and Latoxan (Valence, France). Thus, the commercial available CTX isotoxins can substitute CTX3 for constructing the turn-on fluorescent probe constructed in the present study. Unless otherwise specified, all other reagents were analytical grade.

### 3.1. Sample Preparation and Analysis

All DNA samples and polysaccharides were prepared in 10 mM HEPES (pH 8.0). 5′-FAM/3′-DABCYL-labeled MB (10 nM) and aptamer (20 nM) was incubated with varying concentrations of CTX3 for 5 min at 30 °C, and then titrated with small aliquots of polysaacharides including heparin, chondroitin sulfate and hyaluronic acid. Total dilution never exceeded 10% and the relative fluorescence values were uniformly corrected for dilution. The fluorescence spectra were measured on a Hitachi F-4500 Fluorescence spectrophotometer (Hitachi, Tokyo, Japan) with excitation and emission wavelengths at 480 and 520 nm, respectively.

### 3.2. Analysis of Heparin in Plasma

Human serum was purchased from Sigma-Aldrich Inc. (St. Louis, MO, USA), and the human serum (99 μL) was spiked with heparin solution (1 μL). The spiked samples contained varying heparin concentration (36 μg/mL–1800 μg/mL). Spiked samples (1 μL) were added directly into 2 mL of 10 mM HEPES (pH 8.0) solution containing CTX3-MB or CTX3-aptamer sensors. Then, the fluorescence spectra were measured using excitation and emission wavelengths at 480 and 520 nm, respectively.

### 3.3. Binding of Fluorescein Isothiocyanate (FITC)-Labeled CTX3 with Heparin

CTX3 (0.07 μM/mL) dissolved in 100 mM sodium borate (pH 8.6) was incubated with two-fold molar excess of FITC for 24 h. The FITC-labeled CTX3 (FITC-CTX3) was separated from unlabeled CTX3 by HPLC on a reversed phase SynChropak PR-P column (4.6 mm × 25 cm), which was equilibrated with 0.1% trifluoroacetic acid and eluted with 25–50% acetonitrile. FITC-CTX3 (71.4 nM) was titrated with increasing concentrations of heparin until maximal changes in fluorescence intensity of FITC-CTX3 was achieved. According to the change in the fluorescence intensity, the appropriate heparin concentration was used for incubating with FITC-CTX3. Increasing concentrations of unlabeled CTX3 were then added to compete for binding of FITC-CTX3 with heparin. Competitive binding was monitored at excitation wavelength and emission wavelengths at 550 and 580 nm, respectively. A plot of the 1/(F − Fo) versus 1/[CTX3] gives lines with a slope corresponding to the dissociation constant of CTX3-heparin complexes. Fo and F are fluorescence intensity in the absence or presence of unlabeled CTX3.

### 3.4. Circular Dichroism (CD) Measurement

CD spectra were obtained on a Jasco J-810 spectropolarimeter (JASCO Corporation, Tokyo, Japan) with a cell path-length of 0.5 mm. The CD spectra were measured from 260 nm to 190 nm, and CD spectra were obtained by averaging the signals of five scans.

## 4. Conclusions

In conclusion, the data of the present study suggest that a fluorescent assay was successfully developed for the sensitive and rapid detection of heparin by taking advantage of the competitive binding of CTX3 between an aptamer or MB and heparin. In a HEPES buffer, the low heparin LOD of the CTX3-aptamer and CTX3-MB sensors reached 8.7 ng/mL and 8.0 ng/mL, respectively. The LOD of heparin in serum for the CTX3-aptamer and CTX3-MB sensors can reach 16 ng/mL and 15 ng/mL, respectively. Noticeably, the recommended therapeutic range of heparin in plasma is 17–67 μM (306–1206 μg/mL, MW of heparin ~18,000) during cardiovascular surgery and 1.7–10 μM (30.6–180 μg/mL) during postoperative and long-term care. The detected range (18–360 ng/mL) of CTX3-aptamer and CTX3-MB sensors for heparin can be achieved by dilution of physiological heparin concentration in serum samples. Accordingly, the sensitivity of the two sensor systems developed in the present study is sufficient to detect clinically relevant concentrations of heparin in 1 μL serum samples. Furthermore, the newly constructed sensors are convenient because of the short response time of 5 min and can be used to detect heparin in plasma immediately. In view of the findings that the fluorescence recovery of CTX3-aptamer and CTX3-MB sensors by ChS was lower than that by heparin, it is evident that heparin has a higher capability to detach CTX3 from aptamer and MB compared to ChS. Nevertheless, one may inquire about the sensors not being able to specifically monitor the exogeneous anticoagulants in blood samples owing to endogenous ChS. Previous studies showed that the concentration of ChS ranged from 0.3 to 5.3 μg per mL of human plasma [21]. The serum ChS concentration is about two orders or one order of magnitude below heparin therapeutic or postoperative care concentration, respectively. As shown in supplementary Figure S2, the addition of 1 μL serum containing 30.6 ng heparin (the lowest concentration during postoperative care) or 306 ng heparin (the lowest concentration during surgery) notably recovered FAM fluorescence intensity of CTX3-aptamer and CTX3-MB sensors, but the magnitude of fluorescence recovery was not changed by the addition of 6 ng ChS. These results suggest that the possible interferences of ChS can be excluded during the monitoring of the exogenous heparin.

Compared to the “turn-on” fluorescent A_12_-MB-A_12_/coralyne system reported in previous studies [22], CTX3-MB and CTX3-aptamer sensors are much less efficient in the discrimination of heparin from ChS. However, the MB/coralyne system detects spiked concentration of heparin in human serum samples over the range of 14.4 to 72 μg/mL, and the LOD is 10 μg/mL. It appears that the sensitivity of MB/coralyne for heparin detection is much lower than that of CTX3-MB and CTX3-aptamer sensors. On the other hand, the “turn-on” fluorescence methods using lysine polymer–fluorescence labeled peptide for detecting heparin had also been reported previously [23]. The LOD of lysine polymer-fluorescence labeled peptide for detecting serum heparin is 40.5 μg/ml, and ChS is almost as efficient as heparin in turn-on fluorescence. Zu et al. [24] designed a solid-phase tagging method for enriching heparin from serum, and the LOD of heparin is 25 ng/mL. However, the solid-phase tagging method requires a time-consuming separation procedure and derivation of heparin for fluorescence measurement. Thus, the CTX3-MB and CTX3-aptamer sensors might provide a sensitive and rapid method for detecting heparin. However, it is worth noting that the high sensitivity of the sensors might cause greater error when inaccurate volume of serum is used to analyze heparin. In summary, our data suggest that CTX3-aptamer and CTX3-MB sensors can be used to detect heparin in human serum samples. In addition, this work provides an optimal approach to designing turn-on probes, which can be beneficial for the studies on various other targets that can bind to CTX3. 

## Supplementary Materials

The following are available online. Figure S1: The effect of heparin on the FAM fluorescence of 5′-FAM-and-3′-DABCYL -labeled aptamer or MB; Figure S2: The effect of endogenous ChS concentration on the measurement of heparin using CTX3-aptamer or CTX3-MB sensors.

## Abbreviations

CD	circular dichroism
CTX3	cardiotoxin 3
ChS	chondroitin sulfate
DABCYL	4-([4-(dimethylamino)phenyl]azo)-benzoic acid
FAM	carboxyfluorescein
FITC	fluorescein isothiocyanate
HA	hyaluronic acid
HEPES	4-(2-hydroxyethyl)piperazine-1-ethanesulfonic acid
LOD	limit of detection
MB	molecular beacon
