# Supplementary material for: A Turn-on Fluorescence Sensor for Heparin Detection Based on a Release of Taiwan Cobra Cardiotoxin from a DNA Aptamer or Adenosine-Based Molecular Beacon
Source: Molecules. 2018 Feb 19;23(2):460. doi: 10.3390/molecules23020460 (PMC6017339; doi:10.3390/molecules23020460)
Supplement: Supplementary file 1 [file molecules-23-00460-s001.pdf]

Supplementary Figure Legends

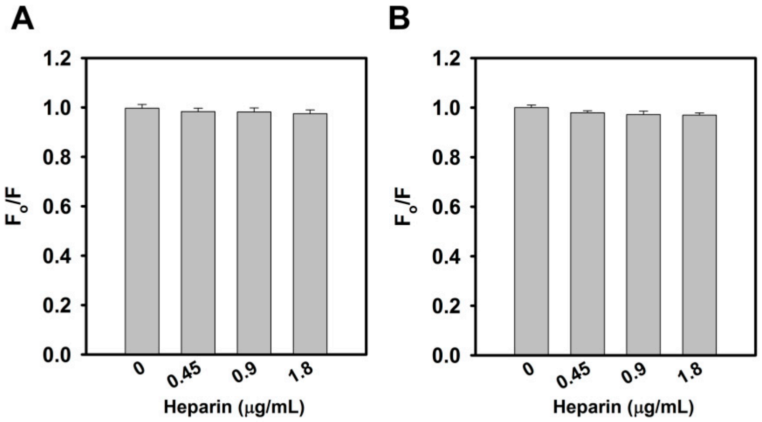

Figure S1. The effect of heparin on the FAM fluorescence of 5′-FAM-and-3′-DABCYL-labeled aptamer or MB. The used aptamer and MB concentrations were 20 nM and 10 nM, respectively. Fluorescence intensity (520 nm) of a solution containing (A) aptamer or (B) MB were measured in the presence of indicated heparin concentrations.

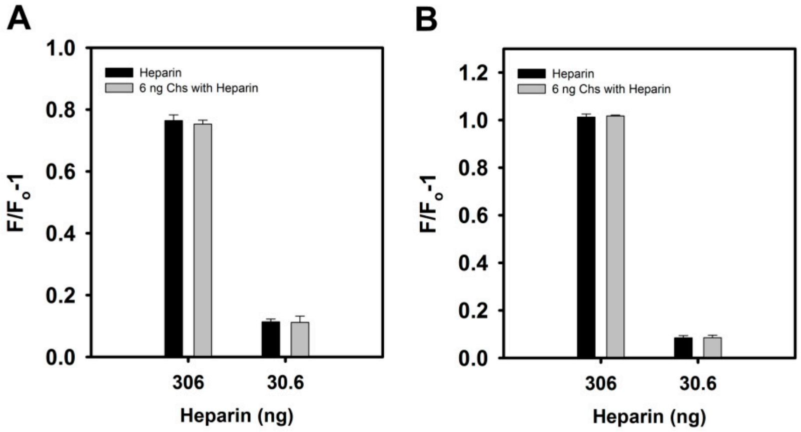

Figure S2. Effect of endogenous ChS concentration on the measurement of heparin using CTX3-aptamer or CTX3-MB sensors. The addition of indicated amounts of heparin (in 1 μL of serum) recovered the fluorescence intensity of CTX3-aptamer or CTX3-MB sensors in 2 mL of 10 mM HEPES (pH 8.0) solution. Addition of 6 ng ChS did not further increase the fluorescence recovery of CTX3-aptamer or CTX3-MB sensors caused by the added heparin.
